# Distance to available services for newborns at facilities in Malawi: A secondary analysis of survey and health facility data

**DOI:** 10.1371/journal.pone.0254083

**Published:** 2021-07-07

**Authors:** Kimberly Peven, Cath Taylor, Edward Purssell, Lindsay Mallick, Clara R. Burgert-Brucker, Louise T. Day, Kerry L. M. Wong, Christabel Kambala, Debra Bick

**Affiliations:** 1 Florence Nightingale Faculty of Nursing, Midwifery & Palliative Care, Kings College London, London, United Kingdom; 2 Maternal and Newborn Health Group, Department of Infectious Disease Epidemiology, London School of Hygiene & Tropical Medicine, London, United Kingdom; 3 School of Health Sciences, University of Surrey, Guildford, United Kingdom; 4 Little Havens Children’s Hospice, Benfleet, United Kingdom; 5 University of Maryland, College Park, MD, United States of America; 6 Avenir Health, Glastonbury, CT, United States of America; 7 RTI International, Washington, DC and London School of Hygiene and Tropical Medicine, London, United Kingdom; 8 Centre for Mathematical Modelling of Infectious Diseases, Department of Infectious Disease Epidemiology, London School of Hygiene and Tropical Medicine, London, United Kingdom; 9 Environmental Health Department, Malawi University of Business and Applied Sciences, Blantyre, Malawi; 10 Warwick Clinical Trials Unit, University of Warwick, Coventry, United Kingdom; Federal University of Sergipe, BRAZIL

## Abstract

**Background:**

Malawi has halved the neonatal mortality rate between 1990–2018, however, is not on track to achieve the Sustainable Development Goal 12 per 1,000 live births. Despite a high facility birth rate (91%), mother-newborn dyads may not remain in facilities long enough to receive recommended care and quality of care improvements are needed to reach global targets. Physical access and distance to health facilities remain barriers to quality postnatal care.

**Methods:**

Using data We used individual data from the 2015–16 Malawi Demographic and Health Survey and facility data from the 2013–14 Malawi Service Provision Assessment, linking households to all health facilities within specified distances and travel times. We calculated service readiness scores for facilities to measure their capacity to provide birth/newborn care services. We fitted multi-level regression models to evaluate the association between the service readiness and appropriate newborn care (receiving at least five of six interventions).

**Results:**

Households with recent births (n = 6010) linked to a median of two birth facilities within 5–10 km and one facility within a two-hour walk. The maximum service environment scores for linked facilities median was 77.5 for facilities within 5–10 km and 75.5 for facilities within a two-hour walk. While linking to one or more facilities within 5-10km or a two-hour walk was not associated with appropriate newborn care, higher levels of service readiness in nearby facilities was associated with an increased risk of appropriate newborn care.

**Conclusions:**

Women’s choice of nearby facilities and quality facilities is limited. High quality newborn care is sub-optimal despite high coverage of facility birth and some newborn care interventions. While we did not find proximity to more facilities was associated with increased risk of appropriate care, high levels of service readiness was, showing facility birth and improved access to well-prepared facilities are important for improving newborn care.

## Introduction

Malawi more than halved its neonatal mortality rate between 1990 and 2019, from 50 to 20 deaths per 1,000 live births [1] and substantially increased the facility birth rate from 55% in 1992 to 91% in 2015–6 [2]. In addition to providing maternal and newborn care free at the point of access, Malawi developed a national newborn action plan (in response to the global Every Newborn Action Plan) to strengthen the continuum of care for women and children [3, 4], defined a newborn mortality reduction target, and implemented a strategy for community engagement/mobilisation for maternal and newborn health [5]. However, the burden of neonatal deaths remains high, with an estimated 12,000 deaths in 2019, the top quartile by country [1]. Given high coverage of facility births, further investigation is needed to understand the quality of care at the time of birth and immediate postpartum period. This avenue of research is supported by global efforts to understand effective coverage, which has been measured as the product of intervention coverage and either input measures/service readiness, service provision/quality of care, or health outcomes achieved [6].

Service readiness is a prerequisite for quality of care; it describes a health facility’s capacity to provide health services and requires components such as basic amenities, basic equipment, and essential medicines. Specific to birth and newborn care this includes equipment such as neonatal bag and masks and essential medicines such as antibiotic eye ointment [7]. World Health Organization (WHO) standards for improving quality of maternal and newborn care in health facilities outlines input measures for quality of facilities including basic essential equipment for labour and childbirth, written up-to-date clinical protocols consistent with WHO guidelines, maternity unit staff receive regular in-service training [8]. In Malawi, primary care facilities (health centres) provide basic emergency obstetric and neonatal care (EmONC) services while secondary (district hospitals) and tertiary (central hospitals) facilities provide comprehensive EmONC services (including surgery and blood transfusion) [9]. A recent assessment of four district hospitals in Southern Malawi showed WHO standards of care were met for laboratories; however, newborn assessments were not completed, monitoring of newborns’ breathing and temperatures was irregular, and documentation was poor [10]. Further improvements in neonatal survival will require improving access to high-quality care and adherence to standards of care around the time of birth [11].

Recommended routine postnatal care for the newborn includes physical assessment for early detection of complications and counselling for women on how best to take care of themselves and their newborns including breastfeeding, counselling on danger signs, umbilical cord care, and temperature monitoring [8, 12]. While WHO recommends remaining in the facility for postnatal care for 24 hours following facility birth, when the most deaths occur [12], women and newborns in Malawi are often discharged earlier due to availability of space and funding. Additionally, facilities do not always prioritise allocating skilled providers for care following uncomplicated births [13]. These shortages of qualified staff as well as essential equipment represent contributors to delayed and incomplete care [14].

Distance and physical access to health facilities remains a major barrier to accessing birth and newborn postnatal care in low- and middle-income countries [15, 16]. Malawi’s small land area and good hospital and road network means hospitals are more accessible [17]; however, a study of maternal deaths in Malawi found over half of the women who died experienced delays due to long distance to a health facility. Additional delays were due to slow transport (e.g. ox cart) and high transport cost [18]. Beyond decision to use health services, having available and equipped services and being able to access them represent additional barriers to appropriate newborn care [14].

### Study aims

This study aims to describe household proximity to health facilities and the readiness of these facilities to provide care around the time of birth and examine the relationship between these features and receipt of newborn care interventions in Malawi, specifically:

1. **Appropriate care**: what is the coverage of recommended newborn care interventions in the first two days of life in Malawi?
2. **Distance to care**: what proportion of households with recent births have a health facility providing care around the time of birth within 5-10km or two-hour travel time?
3. **Service environment:** what level of service readiness do facilities have and is there a relationship between the number of nearby facilities or the service readiness of these facilities and coverage of newborn care?

## Methods

### Ethics

Data for this study were used under an agreement with the DHS Program. The original survey protocol was reviewed and approved by the National Health Sciences Research Committee in Malawi and the ICF Institutional Review Board. Informed consent and voluntary participation were ensured before each interview and data were kept strictly confidential during the survey implementation and identifying information was destroyed after data processing. The King’s College London College Research Ethics Committee granted approval to conduct these analyses (LRS-17/18-5570) and the project has been registered with the King’s College London Data Protection Registration (DPRF-17/18-8170).

### Data

We analysed individual and health facility data from two data sources: the 2013–14 Malawi Service Provision Assessment (SPA) survey and the 2015–16 Malawi Demographic and Health Survey (DHS) [2, 19].

#### Health facility data

SPA surveys collect information on health service availability and the readiness to provide these services [20]. The 2013–14 Malawi SPA was conducted as a census, surveying all facilities in Malawi including all hospitals, health centres, clinics, dispensaries, and health posts. Primary level services include community or rural hospitals, health centres, clinics, dispensaries, health posts, and support for community-based health programs. District hospitals provide inpatient and outpatient care and serve as referral hospitals for primary level facilities. Tertiary services are covered by central hospitals, and serve as referral hospitals for district hospitals, which are situated with secondary level services. Hospitals and health centres are almost exclusively responsible for providing normal birth services [19]. SPA data included information from 977 of the 1060 health facilities in Malawi (other facilities refused participation, were inaccessible, closed, not yet operational, or a respondent was not available).

#### Individual data

DHS surveys collect data through face-to-face interviews with household representatives and women of reproductive age. Complex multistage sampling with stratification is designed to provide representative national estimates of important demographic and health indicators [21]. For the Malawi DHS, 850 standard enumeration areas (SEA) from the 2015–16 Malawi Population and Housing Census were selected with probability proportional to size, independently from 56 sampling strata in the first stage. SEAs which had more than 250 households were split into segments with one segment selected with probability proportional to size such that survey clusters were either an SEA or a segment of an SEA. Following a household listing operation in each cluster, the second stage included selection of a fixed number of households (30 in urban clusters, 33 in rural clusters) using equal probability systematic selection [2].

We selected for inclusion the most recent birth in the two years preceding the survey from the 2015–16 DHS. A priori, newborns were excluded if they were not two days of age at the time of the survey, or had not survived the first two days of life as the interventions of interest included the content of care offered during this initial time period. Newborns who had not yet reached two days of age might yet receive the interventions of interest and some interventions of interest may not have been appropriate for newborns who died soon after birth. We also excluded newborns if the woman reported not living in the current community at the time of the birth.

#### Geography of Malawi

One-third of Malawi’s land area is forest area [22] and about 20% is covered by water, primarily Lake Malawi. The Highlands reach an elevation of 1,600–3,000 metres above sea level [23]. According to the 2018 Malawi Population and Housing Census, the population was 17,563,749 with 12% of the population residing in one of four major cities (Blantyre, Lilongwe, Mzuzu, and Zomba) and another 4% residing in other urban areas [24]. While Malawi has an extensive road network, walking is the most used mode of travel in rural and urban areas [23]. Community studies have shown people travel long distances to access health facilities under difficult terrain in some areas of Malawi [25].

### Variables

#### Facility-level variables (SPA)

The key independent variables reflected proximity to health facilities providing birth services, and readiness to provide birth services of those proximate facilities. We constructed a service readiness score for birth and newborn services with an equal weighting approach similar to that used by Wang et al. [26] and based on the WHO Service Availability Readiness Assessment (SARA) manual [7]. Comparison of measures of quality of care have found this type of weighted additive method to be preferable to simple additive methods or principal components analysis [27].

The score assessed six domains of service readiness comprising: 1) basic emergency obstetric care; 2) newborn signal functions and immediate care; 3) general requirements (e.g. electricity, 24/7 skilled birth attendance); 4) equipment (e.g. neonatal bag and mask); 5) medicines and commodities (e.g. antibiotics); and 6) guidelines (e.g. CEmOC), staff training (e.g. thermal care), and supervision.

Each domain included 4–15 dichotomous indicators (‘yes’ representing availability, ‘no’ representing no availability) which were summed and standardised to have a maximum score of 100 (definitions of all included indicators are presented in S1 Table). The score is interpreted as the percentage of readiness the facility has to provide services. A facility with 100% has a positive response for every measured indicator and a facility with 0% has none of the measured equipment, staff training or other indicators.

#### Individual-level variables (DHS)

The primary outcome measure was receipt of appropriate newborn care. We created a co-coverage index of newborn care interventions, using a method similar to Victora et al. [28] and Carvajal-Aguirre et al. [29], adding the number of care components women reported their newborns had received from six provider-initiated interventions recommended by WHO [12]. We considered newborns who received at least five out of the six interventions to have received appropriate care. This included newborns who received all six interventions (optimal) and those who received any combination of five interventions (pragmatic). The interventions considered included: weighing at birth, mother counselled on breastfeeding, mother counselled on newborn danger signs, breastfeeding episode observed, umbilical cord examined, newborn’s temperature taken. The survey questions for these interventions are presented in S2 Table.

Facility and home births were included in this analysis. A qualitative study of women giving birth outside of facilities in Malawi showed that most in the sample subsequently went to a facility the same day as the birth [30] suggesting that proximity to and quality of facilities should be considered for early newborn care even among home births. While the interventions we included were specifically about delivered by health care providers, we did not distinguish between health care providers delivering interventions in the home/community setting or facility setting nor did we distinguish between pre- or post-discharge for facility births. Additional analysis excluding home births is also presented.

### Linking individual and facility data

DHS surveys collect GPS location points at the centroid of household clusters and SPA surveys collect the GPS location of health facilities. GPS location data for health facilities represent the true location of the facility, however, household cluster data were displaced by the DHS programme prior to release to protect the respondents’ identities (urban clusters up to two kilometres (km), rural clusters up to five km with a further randomly selected 1% displaced up to ten km [31]. We used GPS location data from household clusters to link households to nearby health facilities providing birth/newborn services in Malawi using three methods: distance, travel time with the fastest mode of transport, and walking time.

For each linking method, we categorised the number of facilities linking to households within the specified distance/time into three groups: no facility, one facility, or two or more facilities. Similarly, for each linking method we grouped service environment scores into terciles based on the highest score among all facilities linking with a household. Low, middle and high terciles were chosen to improve interpretation and understanding over use of a continuous score.

#### Distance

**Fig 1:**
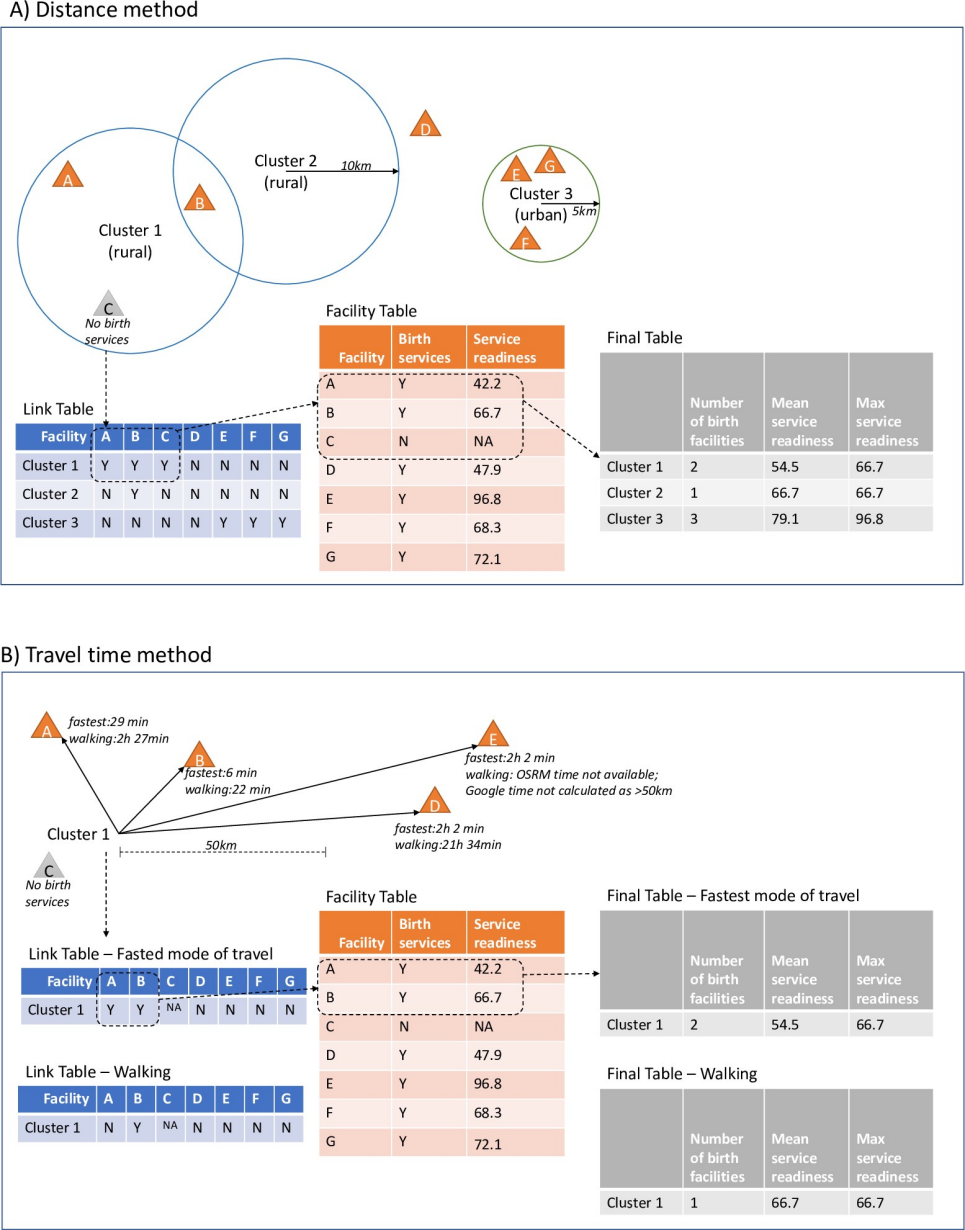
Illustrative example of household cluster and facility linking.

We calculated the straight-line distance between every DHS household cluster and every health facility in Malawi. A Euclidean buffer link method [32–34] was used to create a buffer centred around each DHS cluster using a radius of 5 km in urban areas and 10 km in rural areas to account for displacement of household clusters. All health facilities falling in these 5–10 km buffer areas are considered linked to the household cluster, without consideration of sub-national boundaries. Fig 1A shows an illustrative example of household-facility distance linking.

#### Travel time

We calculated travel time from household clusters to facilities providing birth services using two scenarios: a) fastest possible mode of transportation (i.e. best-case scenario, similar to other studies estimating travel time to hospitals [16, 35, 36]) and b) walking only (i.e. worst-case scenario).

*A) Fastest mode of transportation*. we used the 2015 Malaria Access Project Global Friction Surface [37, 38] which divides the world into one kilometre-square grid cells with each cell value representing the difficulty of crossing the one kilometre cell based on road quality, bodies of water, and sloping terrain. Assuming use of the fastest possible mode of transportation, an algorithm was applied to identify the path requiring the least time to travel between any two points on the friction surface [37, 39]. We employed the algorithm for all possible pairs of DHS household clusters (n = 850) and health facilities with birth services (n = 540), a total of 459,000 pairs. Travel time could not be calculated for 17,360 pairs (3.8%), however, largely for combinations involving one point on Likoma or Chizumulu Island and one point on Malawi mainland as well as for a few health facilities on the national border with Mozambique. All household-facility pairs within a two-hour travel time (fastest mode) were classified as linked.

*B) Walking only*. As many women in Malawi walk to health centres [40], we also calculated travel times for walking as a worst-case scenario. To calculate walking times we used the Open Source Routing Machine (OSRM) API [41] and Google Maps Platform Directions API [42, 43] to plot the optimal route by foot and compute an estimated travel time. We first attempted to calculate walking times for all 459,000 household-cluster-health-facility pairs using OSRM, however, OSRM walking times could not be calculated for 47,381 pairs. For these 47,381 pairs, we calculated the distance and identified pairs <50 km apart (n = 1,775) using the Haversine method which assumes a spherical earth ignoring ellipsoidal effects [44]. Using the Google Maps API, we calculated walking times for 1,742 of the pairs <50 km apart, however walking times could not be calculated for 33 pairs. These totalled 411,619 OSRM walking time estimates (89.7%) and 1,742 Google Maps walking time estimates (0.4%). No walking time estimate was calculated for 45,639 pairs, however all but 33 were determined to be >50km apart. Examination of the coordinate pairs for missing points showed them to be in national parks, forest reserves, or across bodies of water. All household-facility pairs within a two-hour walking time were classified as linked.

Fig 1B shows an illustrative example of household-facility travel time linking.

### Analysis

Simple weighted descriptive statistics on coverage of appropriate care and proximity and service readiness of facilities were calculated. We fitted generalised linear mixed models to examine the relationship between co-coverage of newborn care and the number of linked facilities or the service environment of linked facilities. To estimate risk ratios for our binary outcome variable, we used a Poisson distribution with a logarithm link function and robust standard errors [45]. We controlled for socio-demographic and birth-related factors (population density, place of birth (home/health facility), wealth quintile (DHS-provided), maternal age at birth, and maternal education). Population density was obtained from The DHS Program’s Spatial Data Repository Geospatial Covariates which uses the average United Nations population density within the surrounding buffer area (2 km for urban clusters or 10 km for rural) [46]. We grouped households into terciles based on population density.

As individuals are nested within clusters (SEAs) and our main predictors are cluster-level variables, the multilevel models account for this nesting and simultaneously test the effects of cluster-level and individual-level predictors on the individual outcome. While covariates had fixed effects, intercepts could vary randomly across clusters. Level-1 and level-2 weights were computed using a method described by Elkasabi et al. where level-1 individual weights are denormalised and the level-2 cluster weights are approximated by equally allocating the variation between the individual and cluster levels (α = 0.5) [47].

All geographic linking and descriptive statistical analyses were conducted in R [48], using the survey package [49] to adjust for the complex sampling design. DHS-provided weights were used to account for sampling probability and non-response. Multilevel models were fitted in STATA 16 using the *svy*: *melogit* command [50].

## Results

### Health facilities and service environment

**Fig 2:**
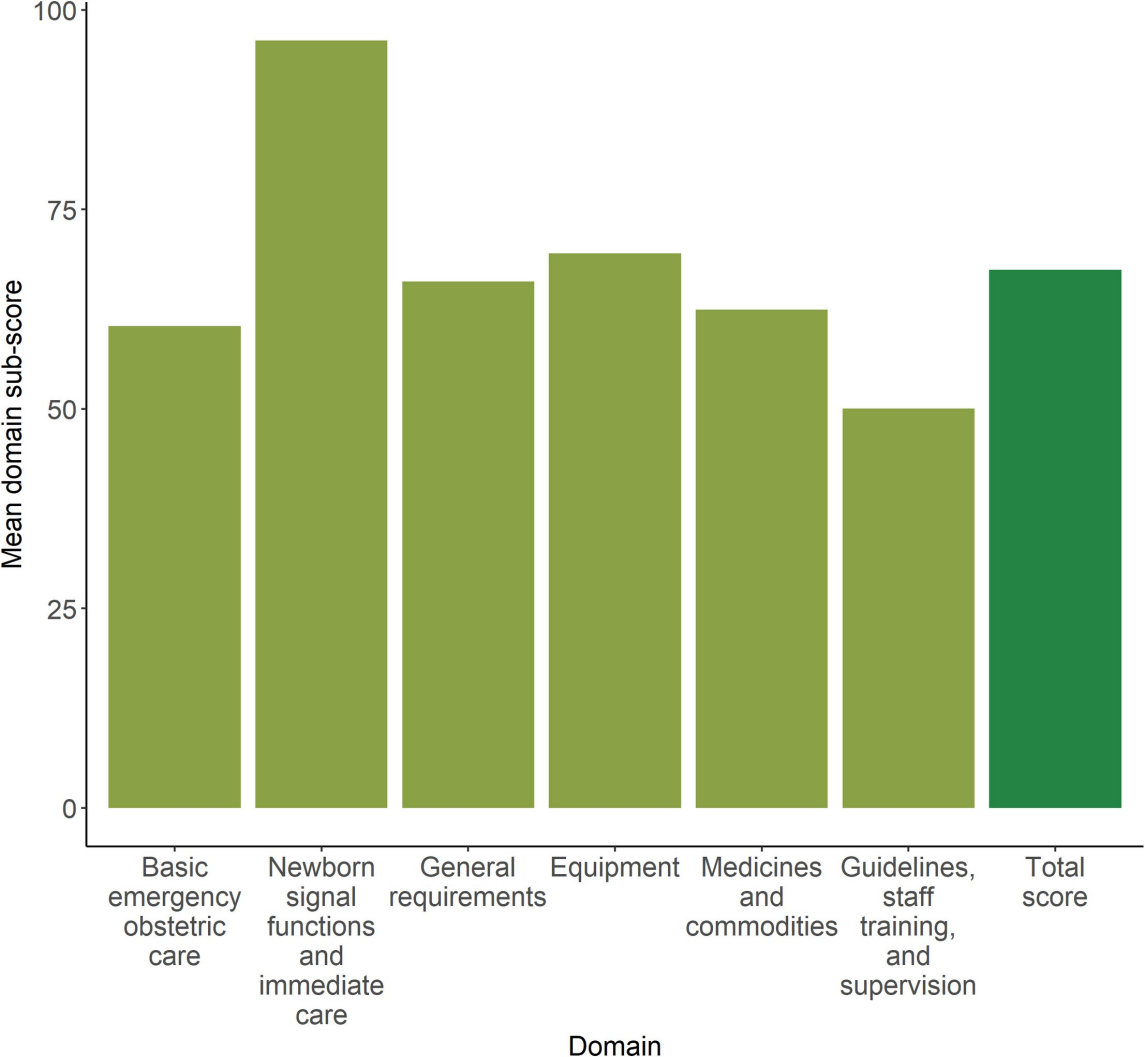
Mean service readiness scores and domain sub-scores (and 95%Cis) for facilities providing care around the time of birth (n = 540).

Among the 540 (528 weighted facilities) facilities reporting providing care around the time of birth, service readiness ranged from 27.5 to 98.7% (mean = 67.4, median = 67.2, IQR = 59.3–75.6). Mean domain sub-scores ranged from 50.1% for the guidelines, staff training, and supervision domain to 96.2% for the newborn signal functions and immediate care domain (Fig 2). Few facilities had improved sanitation (24.9%). Most facilities had disposable latex gloves (97.4%) and at least one birth bed (98.5%). Additionally, most facilities reported having routinely practicing skin-to-skin (98.1%), breastfeeding in the first hour (98.9%), and drying and wrapping newborns (99.8%).

### Household sample characteristics and newborn care co-coverage

Of the 6567 women who had their most recent births in the two years prior to the survey, 6010 reported residing in the same village/town/city since the time of the birth and were included in the analysis. 12.5% of births were in urban areas, 20.0% were to women under age 20, and 19.6% were to women who attended secondary or higher education (Table 1). Of the 412 births (6.9%) that took place outside of facilities (e.g. at home), a smaller proportion were urban residents (6.9%), more than half were in the poorest two wealth quintiles (63.4%), and a smaller proportion had secondary or higher education (6.5%).

**Table 1:** Background characteristics for the most recent births for local area residents in the two years preceding the survey.

	*All births (n = 6010)*	*Facility births (n = 5598)*	*Non-facility births (n = 412)*
*Characteristic*	n (%)	n (%)	n (%)
*Residence*			
*Urban*	754 (12.5)	726 (13.0)	29 (6.9)
*Rural*	5256 (87.5)	4873 (87.0)	383 (93.1)
*Population density*			
*Lowest*	1587 (26.4)	1468 (26.2)	119 (28.9)
*Middle*	2239 (37.3)	2097 (37.5)	142 (34.5)
*Highest*	2183 (36.3)	2032 (36.3)	151 (36.6)
*Wealth quintile*			
*Poorest*	1557 (25.9)	1409 (25.2)	148 (36.0)
*Poorer*	1399 (23.3)	1286 (23.0)	113 (27.4)
*Middle*	1179 (19.6)	1096 (19.6)	83 (20.2)
*Richer*	1008 (16.8)	961 (17.2)	47 (11.5)
*Richest*	867 (14.4)	846 (15.1)	21 (5.0)
*Previous live births*			
*First live birth*	1563 (26.0)	1500 (26.8)	63 (15.3)
*Second order birth or higher*	4448 (74.0)	4099 (73.2)	349 (84.7)
*Mother’s age at birth (years)*			
*<20*	1203 (20.0)	1132 (20.2)	72 (17.4)
*20–34*	4045 (67.3)	3789 (67.7)	257 (62.3)
*35+*	762 (12.7)	678 (12.1)	84 (20.3)
*Education*			
*No education or primary*	4833 (80.4)	4447 (79.4)	385 (93.5)
*Secondary or higher*	1178 (19.6)	1151 (20.6)	27 (6.5)

**Fig 3:**
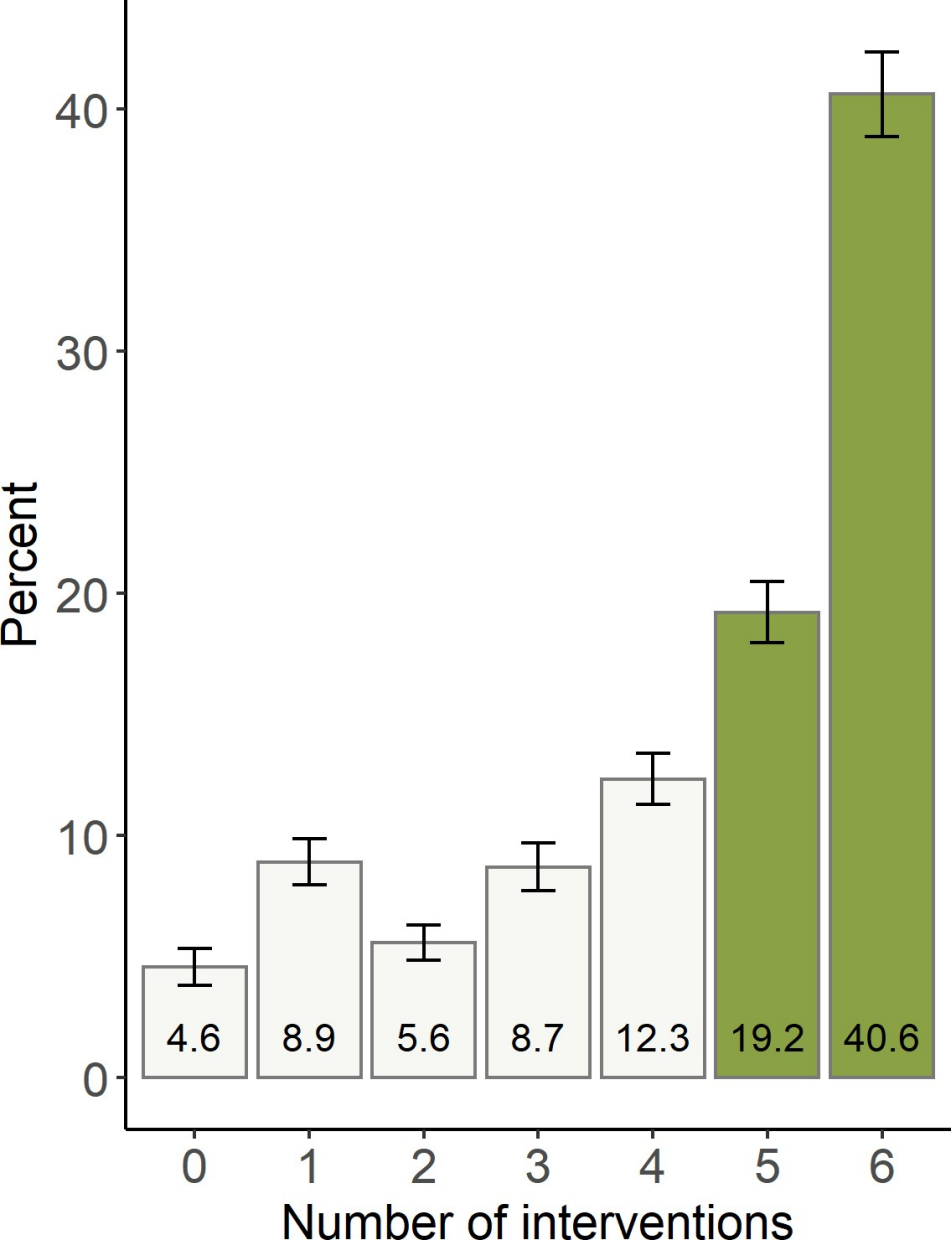
Co-coverage of newborn care.

Just over 95% of newborns received at least one of the six early newborn care intervention of interest however, fewer than two-thirds (59.8%) received appropriate care (co-coverage of five or six interventions) (Fig 3). Of newborns born at home far fewer received appropriate care (19.1%) and almost half (45.8%) did not receive any interventions in the first two days (S3 Table).

### Linked analysis

#### Distance

**Fig 4:**
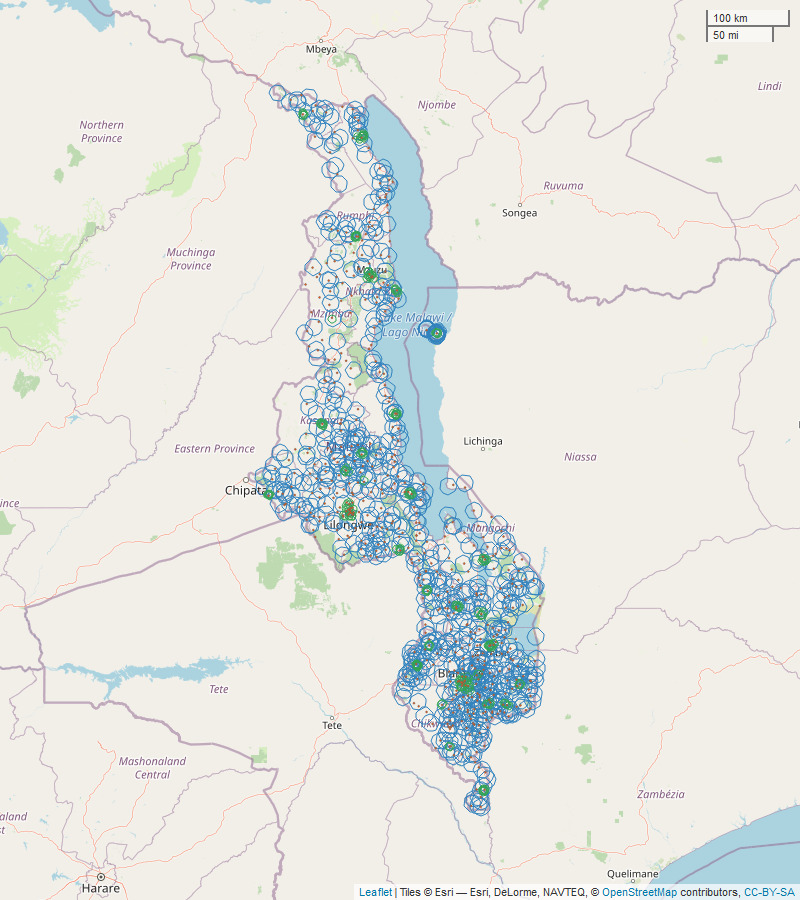
Map of Malawi, distance to facilities. Blue circles represent the 10km buffer area around rural clusters, green circles represent the 5km buffer area around urban clusters, red dots represent health facilities providing birth services. Base map and data from OpenStreetMap and OpenStreetMap Foundation.

Fig 4 contains a map showing the household clusters with included newborns and health facilities providing birth services. Of the 540 facilities with birth services, 509 linked to household clusters in the DHS survey and had a similar distribution of service readiness (median = 67.2, IQR = 59.4–75.6).

Recent births linked to a median of two facilities (IQR:1,3) with birth services (Table 2). While 4.4% of births (n = 264 weighted births in 35 of 850 clusters) did not link to any facility within the specified 5km urban or 10km rural distance, some linked to as many as 12 facilities (n = 8 weighted births in one household cluster). Among births in clusters with linked facilities, the maximum service environment score among linked facilities median was 77.5 (IQR:69.2,85.3), lower among home births (median:75.5; IQR:65.3,83.4).

**Table 2:** Distance, travel time, and service environment scores for linked facilities.

	*All births*	*Facility births*	*Non-facility births*
***Number of facilities***	n (%)	n (%)	n (%)
*5-10km distance*			
*No facility*	264 (4.4)	223 (4.0)	41 (9.9)
*One facility*	1559 (25.9)	1469 (26.2)	90 (21.8)
*Two or more*	4187 (69.7)	3906 (69.8)	281 (68.3)
*Median (IQR)*	2 (1,3)	2 (1,3)	2 (1,3)
*Within two hours (fastest mode)*			
*No facility*	38 (0.6)	28 (0.5)	11 (2.6)
*One facility*	24 (0.4)	20 (0.4)	3 (0.8)
*Two or more*	5948 (99.0)	5550 (99.1)	398 (96.6)
*Median (IQR)*	108 (58,152)	109 (58.2,153)	94 (45.7,136)
*Within two hours walking time*			
*No facility*	1071 (17.8)	960 (17.1)	111 (27.0)
*One facility*	2224 (37.0)	2078 (37.1)	146 (35.3)
*Two or more*	2716 (45.2)	2560 (45.7)	155 (37.7)
*Median (IQR)*	1 (1,2)	1 (1,2)	1 (0,2)
***Travel time to nearest facility (minutes)***	median (IQR)	median (IQR)	median (IQR)
*Within two hours (fastest mode)*	9.6 (4.3,17.6)	9.4 (4.1,17.6)	12.2 (6.7,19)
*Within two hours walking time*	60.7 (36.2,85.4)	59.4 (35.0,84.4)	74.3 (47.9,97.2)
***Service environment score (maximum linked score)***	median (IQR)	median (IQR)	median (IQR)
*5-10km distance*	77.5 (69.2,85.3)	77.5 (69.6,85.3)	75.5 (65.3,83.4)
*Within two hours (fastest mode)*	98.7 (96.6,98.7)	98.7 (96.6,98.7)	98.7 (96.6,98.7)
*Within two hours walking time*	73.1 (64.8,84.1)	73.2 (64.8,84.1)	71.1 (62.5,80.4)

Having one facility or two or more facilities within 5-10km was not associated with an increase in the risk of reporting appropriate newborn care (co-coverage of at least 5 interventions) compared to having no facility (Table 3). Birth outside of a facility was associated with a great decrease in the risk of appropriate newborn care (ARR = 0.32, 95%CI = 0.25,0.41). Living in the most population-dense areas (compared to the least-dense; ARR = 1.09, 95%CI = 1.00,1.19) and secondary or higher maternal education (ARR = 1.08, 95%CI = 1.02,1.15) was associated with an increase in risk of appropriate newborn care in the model with number of linked facilities. However, in the model with service environment scores, only maternal education was still significant (ARR = 1.08, 95%CI = 1.01,1.14). Being within 5-10km of a facility with a middle (ARR = 1.17, 95%CI = 1.06,2.28) or high (ARR = 1.24, 95%CI = 1.13,1.36) service environment score was associated with 1.17–1.24 times the risk of reporting appropriate newborn care with facility birth remaining an important factor (home birth ARR = 0.32, 95%CI = 0.25,0.42).

**Table 3:** Distance linked facility number and service environment association with newborn care co-coverage.

**5-10km Distance**					
**Number of linked facilities**			**Service environment**		
	*ARR*	*95%CI*		*ARR*	*95%CI*
**Number of linked facilities (ref = none)**			**Service environment score (ref = lowest)**		
One facility	0.99	0.82,1.2	No facility (within 5-10km)	1.10	0.91,1.32
Two or more facilities	1.02	0.85,1.22	Middle	**1.17**	**1.06,1.28**
			Highest	**1.24**	**1.13,1.36**
**Home birth**	**0.32**	**0.25,0.41**	**Home birth**	**0.32**	**0.25,0.42**
**Population density (ref = lowest density)**			**Population density (ref = lowest density)**		
Middle density	1.02	0.94,1.11	Middle density	1.00	0.92,1.08
Most dense	**1.09**	**1,1.19**	Most dense	1.02	0.93,1.12
**Wealth (ref = poorest)**			**Wealth (ref = poorest)**		
Poorer	1.06	0.98,1.14	Poorer	1.05	0.97,1.14
Middle	1.07	0.99,1.16	Middle	1.07	0.99,1.16
Richer	1.07	0.99,1.16	Richer	1.06	0.97,1.15
Richest	1.08	0.99,1.19	Richest	1.05	0.96,1.16
**Maternal age at birth (ref = less than 20 years)**			**Maternal age at birth (ref = less than 20 years)**		
20–34 years	0.99	0.93,1.06	20–34 years	0.99	0.93,1.07
35+ years	1.03	0.95,1.13	35+ years	1.04	0.95,1.14
**Maternal education, secondary or higher**	**1.08**	**1.02,1.15**	**Maternal education, secondary or higher**	**1.08**	**1.01,1.14**
Number of births	5958		Number of births	5887	
Number of clusters	837		Number of clusters	828	
**Travel time: 2-hour walk**					
**Number of linked facilities**			**Service environment**		
	*ARR*	*95%CI*		*ARR*	*95%CI*
**Number of linked facilities (ref = none)**			**Service environment score (ref = lowest)**		
One facility	0.94	0.85,1.03	No facility (within 2hr walk)	**1.16**	**1.05,1.29**
Two or more facilities	0.92	0.84,1.02	Middle	**1.11**	**1.01,1.23**
			Highest	**1.16**	**1.05,1.27**
**Home birth**	**0.32**	**0.24,0.41**	**Home birth**	**0.32**	**0.24,0.41**
**Population density (ref = lowest density)**			**Population density (ref = lowest density)**		
Middle density	1.04	0.96,1.14	Middle density	1.03	0.95,1.12
Most dense	**1.12**	**1.03,1.23**	Most dense	1.08	0.99,1.19
**Wealth (ref = poorest)**			**Wealth (ref = poorest)**		
Poorer	1.06	0.98,1.14	Poorer	1.06	0.98,1.14
Middle	1.07	0.99,1.16	Middle	1.07	0.99,1.16
Richer	1.07	0.99,1.17	Richer	1.07	0.99,1.16
Richest	1.09	0.99,1.19	Richest	1.06	0.97,1.17
**Maternal age at birth (ref = less than 20 years)**			**Maternal age at birth (ref = less than 20 years)**		
20–34 years	0.99	0.93,1.06	20–34 years	0.99	0.93,1.06
35+ years	1.03	0.95,1.13	35+ years	1.04	0.95,1.13
**Maternal education, secondary or higher**	**1.08**	**1.02,1.15**	**Maternal education, secondary or higher**	**1.08**	**1.02,1.14**
Number of births	5958		Number of births	5940	
Number of clusters	837		Number of clusters	835	

Crude risk ratios presented in S4 Table.

#### Travel time

Using the fastest mode of transportation, most births (99.0%) had two or more facilities within a two-hour travel time. When considering only walking time, 45.2% of births had a facility within a two-hour travel time. For home births this was slightly lower, 96.6% of home births with a facility within a two-hour travel time using the fastest method, and 37.7% using walking time. The median travel time to the nearest facility within two hours using the fastest mode of transportation was 9.6 minutes for all births, 9.4 minutes for facility births, and 12.2 minutes for home births. Using the walking time to the nearest facility within two hours was 60.7 minutes for all births, 59.4 minutes for facility births, and 74.3 minutes for home births.

Among births in clusters with linked facilities, the median of the maximum service environment scores among linked facilities within two hours (fastest mode) was 98.7 (IQR: 96.6,98.7), the same for all births, facility births, and home births. Considering only walking time the maximum service environment score among linked facilities within two hours was 73.1 (IQR:64.8,84.1) for all births and slightly lower for home births (median:71.1 IQR:62.5,80.4).

Similar to the model for number of facilities within 5-10km, having one facility or two or more facilities within a two-hour walking time was not associated with an increase in risk of reporting appropriate newborn care compared to having no facility (Table 3). Similar to the model for service environment scores of facilities within 5-10km, being within a two-hour walk of a facility with a middle (ARR = 1.11, 95%CI = 1.01,1.23) or high (ARR = 1.16, 95%CI = 1.05,1.27) service environment score was associated with a 11–16% increase in risk of reporting appropriate newborn care compared to a low score (lowest tercile). Additionally, having no facility within a two-hour walk was associated with an increase in risk of reporting appropriate newborn care (ARR = 1.16, 95%CI = 1.05,1.29) compared with a facility with a score in the lowest tercile.

Control variables showed similar risk ratios and confidence intervals to the distance model.

Results excluding home births are presented in S5 Table, no important differences are noted.

## Discussion

This study found that most births in Malawi took place in a community with a facility providing birth services within 5-10km. While choice of facility was wide within a two-hour travel time using the fastest mode of transport, choice (number of facilities) was limited within 5–10 km or two-hour walking time, as was facility readiness to provide services. Although facility birth was the most important factor associated with receiving appropriate newborn care, higher levels of service readiness were an important predictor of appropriate care. These results should be interpreted with caution due to random displacement of the GPS location of households to protect the identity of respondents which may introduce random misclassification [33].

Malawi is a regional leader in terms of high rates of facility birth as well as higher levels of service readiness with limited subnational variation [26, 51]. Additionally, other research has shown relatively high access to hospitals or delivery facilities [16, 17]. Over the last two decades, Malawi has implemented several policies to increase facility birth and improve maternal and newborn health using both supply-side incentives and demand-side disincentives. Initiatives have focused on increasing skilled staff and expanding maternity waiting homes. A ban on traditional birth attendants was implemented in the mid-2000s but even when it was reversed at the national level, women continued to give birth in health facilities as community-level bylaws continued to prohibit traditional birth attendants [52]. Essential services, including childbirth and newborn care, are free at public health facilities (and some non-government facilities with service-level agreements) but it should be noted families often face cost of transport and charges for commodities, food, or items women are meant to provide (e.g. sheets) [53].

Effective coverage (a combination of need, use, and quality of care) research has pointed to the need to consider service readiness in conjunction with service or quality. One study has shown, despite high levels of facility births, after adjusting for the readiness of facilities to provide birth care, the input-adjusted effective coverage (the product of crude coverage times service readiness) yielded a reduction of crude coverage from 93% to 66% [26]. Similar gaps in quality of care and subsequent reductions in crude coverage were identified for other service areas including family planning, antenatal care, and sick child care [54].

Even with high rates of facility births, women and newborns often do not remain in facilities long enough to receive adequate postnatal care [55]. In Malawi, fewer than half of women (47%) and just over two-thirds (68%) of newborns had a postnatal check before discharge from facility birth [56]. Given that postnatal facility time and pre-discharge checks may be limited, our findings of inadequate coverage of appropriate newborn care are not surprising. Carvajal-Aguirre et al. [29] showed that among facility births, facility service readiness was associated with increased odds of receiving appropriate newborn care and practices. This study examined immediate newborn care interventions such as breastfeeding within an hour of birth, no prelacteal feeding, being wiped/dried after birth, and not being bathed in the first six hours. In contrast, in this study we included interventions in a broader time frame (all within the first two days of birth except for weighing at birth) to also examine the association of facility proximity and service readiness among those born at home who may not have accessed care immediately or those who were discharged from facilities without appropriate care. Further, we looked specifically at health care provider initiated interventions, excluding family-led practices such as early initiation of breastfeeding and no prelacteal feeding which could occur without contact with the health system.

Previous studies have shown availability of health facilities is associated with contact coverage of maternal and newborn postnatal care in Malawi [57] and that service readiness is associated with quality newborn care [29]. Similarly, we found an association between service readiness and appropriate newborn care. We know, however, there is a gap between facility delivery/postnatal contact and content/quality of care [58]. Previous studies in Malawi have not examined the relationship between service availability and quality newborn care, we found the number of nearby facilities (service availability) was not significantly associated with appropriate newborn care. This suggests that while availability of services may improve contact coverage, quality-coverage gaps exist that may narrow with improved service quality.

Bhutta et al. [59] showed that closing the quality gap for facility births was important for improving care and newborn survival. Our study identifies a quality gap in newborn care in Malawi. Bhutta et al. [59] outline interventions for quality improvement including audit and feedback mechanisms, training care providers, paying for performance, information systems, social support, and breastfeeding support. However, wider investment is needed, not just relevant to the health system, but education and other infrastructure [60]. Facility-based bottlenecks include staff shortages and crowded facilities, particularly in light of the dramatic increase in facility birth over the past two decades [61]. Performance incentive programmes implemented in some districts in Malawi have been shown to help overcome these challenges and improve maternal and neonatal health service quality [62].

### Strengths and limitations

A strength of this study is that we were able to link households and health facilities using data from the most recent Malawi SPA as it was a census of health facilities. Service readiness measures used in the study contained a wide range of infrastructure, equipment, staff, and signal functions identified by WHO as essential for providing birth and newborn services. We examined co-coverage of newborn care, as facility birth and a postnatal contact are not indicative of recommended content of care.

Some limitations should be noted. While the period during which the service environment was assessed (2013–2014) overlaps with the time period of the births in this study (2013–2016), in some cases more than one year elapsed between the SPA data collection and DHS survey birth. Some bias may have been introduced if the service readiness changed greatly between these two events. Number of facilities and service readiness measures reflect the area in which women lived, however women may have travelled outside of their area to give birth, notably to a referral centre in cases of known pregnancy complications. Additionally, a woman might not attend her closest facility in favour of a preferred facility farther away. Where no facility was identified within walking distance, a facility may be present but walking time was not possible to calculate with our methods. Furthermore, due to the displacement of household cluster GPS data, the nearest identified facility may not be the closest or easiest to reach facility to the true location of the household. DHS displacement processes to protect respondent identity produce approximately uniform distribution of displacement with an average distance of 1km in urban areas and 2.5km in rural areas where points are not displaced beyond administrative boundaries [31]. To limit misclassification bias we used a dataset including a full census of health facilities as using sample surveys of health facilities instead of a census can increase misclassification. Additionally, we avoided direct linking with the closest facility and instead used ecological methods and service environment among all linked facilities as recommended by Skiles et al. [33].

Survey-based measurement of early newborn care interventions is subject to questions being understandable to respondents, respondents having witnessed or been told about the interventions, and being able to accurately report the information at the time of the survey. Qualitative research in Malawi has shown women’s recall of timing of events around the time of birth becomes less precise over time [63]. To increase the likelihood of accurate recall of interventions, we limited the study population to the most recent birth in the two years before the survey. Validation studies of survey-report for interventions around the time of birth have shown mixed results of accuracy of women’s report, even in exit-survey. A study of facility exit survey-reported measurement of birthweight in Bangladesh, Nepal and Tanzania found high sensitivity and moderate specificity compared to observed gold standard [64], other maternal and newborn indicators varied widely by country [65]. In a validity study in Kenya and Swaziland, counselling on danger signs in the newborn had moderate sensitivity and specificity, meeting cut offs for population-level validity but not individual-level validity [66]. Although it did not include validation of the specific interventions in this study, a validation study of maternal and newborn care in Kenya showed that indicators reported accurately at baseline were reported again with accuracy 13–15 months later [67]. While recall bias is an important limitation to consider, survey report remains the main source of population-level data on care around the time of birth.

## Conclusion

While there is high coverage of facility birth and some interventions in the first two days of life, appropriate care remains sub-optimal and women’s choice of nearby facilities is limited. Although we did not find proximity to more facilities was associated with increased risk of appropriate newborn care, high levels of service readiness were, showing that both facility birth and improved access to well-prepared facilities are important for improving newborn care. Malawi is a regional leader in facility birth but quality of care in facilities needs improving. While improved access to care and transport links are important, improving quality at health facilities is essential to reaching global newborn survival goals.

## Supporting information

S1 Table Service readiness score domains and indicators (adapted from Wang et al (2019)). (DOCX)

S2 Table Postnatal care interventions and question wording from the phase seven DHS model questionnaire. (DOCX)

S3 Table Co-coverage of newborn care by place of birth. (DOCX)

S4 Table Crude risk ratios. (DOCX)

S5 Table Sensitivity analysis excluding home births. (DOCX)
