# Supplementary material for: Distance to available services for newborns at facilities in Malawi: A secondary analysis of survey and health facility data
Source: PLoS One. 2021 Jul 7;16(7):e0254083. doi: 10.1371/journal.pone.0254083 (PMC8263259; doi:10.1371/journal.pone.0254083)
Supplement: S1 Table — (DOCX) [file pone.0254083.s001.docx]

S1 Table. Service readiness score domains and indicators (adapted from Wang et al (1))

|  | Indicator | Definition | Proportion of facilities with element and mean domain sub-scores (n=528 weighted facilities) |
| --- | --- | --- | --- |
| Domain A: Basic emergency obstetric care | | | **60.4** |
|  | Parenteral administration of antibiotics | Facility performed this signal function for emergency obstetric care at least once during the three months before the assessment | 81.6 |
|  | Parenteral administration of uterotonic drugs/oxytocin | Facility performed this signal function for emergency obstetric care at least once during the three months before the assessment | 98 |
|  | Parenteral administration of anticonvulsants for hypertensive disorders of pregnancy | Facility performed this signal function for emergency obstetric care at least once during the three months before the assessment | 49.4 |
|  | Manual removal of placenta | Facility performed this signal function for emergency obstetric care at least once during the three months before the assessment | 43.1 |
|  | Assisted vaginal birth | Facility performed this signal function for emergency obstetric care at least once during the three months before the assessment | 51.3 |
|  | Removal of retained products | Facility performed this signal function for emergency obstetric care at least once during the three months before the assessment | 38.9 |
| Domain B: Newborn signal functions and immediate care | | | **96.2** |
|  | Neonatal resuscitation | Facility reports performing neonatal resuscitation at least once during the three months before the assessment | 87.9 |
|  | Skin-to-skin | Facility reports this intervention is routinely practiced | 98.1 |
|  | Breastfeeding in the first hour | Facility reports this intervention is routinely practiced | 98.9 |
|  | Drying and wrapping newborns | Facility reports this intervention is routinely practiced | 99.8 |
| Domain C: General requirements | | | **65.9** |
|  | Electricity | Facility is connected to a central power grid and there has not been an interruption in power supply lasting for more than two hours at a time during normal working hours in the seven days before the assessment, or the facility had a functioning generator with fuel available on the day of the assessment, or else facility has a backup solar power. | 66.4 |
|  | Improved water source | Facility has an improved water source available. For most countries, this means that water is piped into the facility or onto facility grounds, or else water comes from a public tap or standpipe, a tube well or borehole, a protected dug well, protected spring, rain water, or bottled water, and the outlet from this source is within 500 meters of the facility | 95 |
|  | Improved sanitation | Facility has a functioning flush or pour-flush toilet, a ventilated improved pit latrine, or composting toilet | 24.9 |
|  | 24/7 skilled birth attendance | Provider of birth care available on-site or on-call 24 hours/day, with observed duty schedule. | 53.8 |
|  | Emergency transport | The facility had a functioning ambulance or other vehicle for emergency transport that was stationed at the facility and had fuel available on the day of the assessment, or the facility has access to an ambulance or other vehicle for emergency transport that is stationed at another facility or that operates from another facility | 89.5 |
| Domain D: Equipment | |  | **69.4** |
|  | Sterilization equipment | Facility reports that some instruments are processed in the facility and the facility has a functioning electric dry heat sterilizer, a functioning electric autoclave, or a non-electric autoclave with a functioning heat source available somewhere in the facility | 30.8 |
|  | Delivery bed | At least one delivery bed available and observed in delivery area | 98.5 |
|  | Examination light | Examination light (flashlight okay) available, observed, and functioning in delivery area | 31.7 |
|  | Delivery pack | Delivery pack OR cord clamp, episiotomy scissors, scissors/lade to cut cord, suture material with need, AND needle holder all available in delivery area | 92.2 |
|  | Suction apparatus (mucus abstractor) | Suction apparatus (mucus abstractor) available, observed, and functioning in the delivery area | 63 |
|  | Manual vacuum extractor | Manual vacuum extractor available, observed, and functioning in the delivery area | 40.7 |
|  | Vacuum aspirator or D&C kit | Vacuum aspirator or D&C kit available, observed, and functioning, in the delivery area | 24.1 |
|  | Partograph | Partograph available and observed in delivery area | 88.1 |
|  | Disposable latex gloves | Disposable latex gloves observed in delivery area | 97.4 |
|  | Newborn bag and mask | Newborn bag and mask available, observed, and functioning in the delivery area | 89.4 |
|  | Infant scale | Infant scale observed and functioning in delivery area | 95.2 |
|  | Blood pressure apparatus (digital or manual) | Manual or digital blood pressure apparatus observed and functioning in delivery area | 75.6 |
|  | Hand washing soap and running water or hand disinfectant | Hand-washing soap and running water or hand disinfectant available and observed in delivery area | 75.2 |
| Domain E: Medicines and commodities | | | **62.5** |
|  | Injectable antibiotic | Injectable antibiotics observed in delivery area (i.e., at “service site”) and at least one dose valid. | 55.5 |
|  | Hydrocortisone available at the facility | Hydrocortisone observed at the facility and at least one dose valid | 13.1 |
|  | Injectable uterotonic | Oxytocin observed in delivery area with at least one dose valid | 95.2 |
|  | Skin disinfectant | Skin disinfectant other than chlorhexidine | 55.2 |
|  | Magnesium sulphate | Magnesium sulphate available in delivery area with at least one dose valid | 84.5 |
|  | IV solution with infusion set | IV solution with infusion set available in delivery area with at least one set valid | 67 |
|  | Chlorhexidine for cord cleaning | Chlorhexidine solution (4%) for umbilical cord cleaning available in delivery area, with at least one dose valid | 35.8 |
|  | Antibiotic eye ointment for newborn | Tetracycline eye ointment for newborn available in delivery area and at least one dose valid | 93.4 |
| Domain F: Guidelines, staff training, and supervision | | | **50.1** |
|  | Guidelines: Integrated Management of Pregnancy and Childbirth (IMPAC) guidelines | Guidelines available in delivery area | 44.7 |
|  | Guidelines: CEmOC guidelines | Guidelines available in delivery area | 27.4 |
|  | Guidelines: Guidelines for management of pre-term labour | Guidelines available in delivery area | 41.7 |
|  | Training in neonatal resuscitation | At least one provider of delivery/newborn care in facility received training in neonatal resuscitation in the past 24 months | 62.8 |
|  | Training in early and exclusive breastfeeding | At least one provider of delivery/newborn care in facility received training in early and exclusive breastfeeding in the past 24 months | 48.5 |
|  | Training in newborn infection management (including injectable antibiotics) | At least one provider of delivery/newborn care in facility received training in newborn infection management (including injectable antibiotics) in the past 24 months | 40.6 |
|  | Training in thermal care | At least one provider of delivery/newborn care in facility received training in thermal care in the past 24 months | 54.6 |
|  | Training in cord care | At least one provider of delivery/newborn care in facility received training in cord care in the past 24 months | 55.5 |
|  | Training in Kangaroo Mother Care (KMC) | At least one provider of delivery/newborn care in facility received training in KMC in the past 24 months | 40.6 |
|  | Supervision | At least half of interviewed providers reported being personally supervised at least once during the 6 months preceding the survey | 83.9 |

1. Wang W, Mallick L, Allen C, Pullum T. Effective coverage of facility delivery in Bangladesh, Haiti, Malawi, Nepal, Senegal, and Tanzania. Rahman M, editor. PLoS ONE. 2019 Jun 11;14(6):e0217853.
