# Supplementary material for: Distance to available services for newborns at facilities in Malawi: A secondary analysis of survey and health facility data
Source: PLoS One. 2021 Jul 7;16(7):e0254083. doi: 10.1371/journal.pone.0254083 (PMC8263259; doi:10.1371/journal.pone.0254083)
Supplement: S2 Table — (DOCX) [file pone.0254083.s002.docx]

S2 Table. Postnatal care interventions and question wording from the phase seven DHS model questionnaire(1).

| **Intervention** | **Question** | **Proportion with intervention (n=6010)** |
| --- | --- | --- |
| **Weighed at birth** | 427. Was (NAME) weighed at birth? | 86.6 |
| **Umbilical cord check** | 457 a) During the first two days after (NAME)’s birth, did any health care provider do the following: Examine the cord? | 60.5 |
| **Temperature measurement** | 457 b) During the first two days after (NAME)’s birth, did any health care provider do the following: Measure (NAME)’s temperature? | 65.1 |
| **Danger sign counselling** | 457 c) During the first two days after (NAME)’s birth, did any health care provider do the following: Counsel you on danger signs for newborns? | 69.9 |
| **Breastfeeding counselling** | 457 d) During the first two days after (NAME)’s birth, did any health care provider do the following: Counsel you on breastfeeding? | 83.3 |
| **Breastfeeding observation** | 457 e) During the first two days after (NAME)’s birth, did any health care provider do the following: Observe (NAME) breastfeeding? | 70.2 |

1. DHS. DHS Model Questionnaire - Phase 7 [Internet]. Rockville, Maryland, USA: ICF; 2015. Available from: https://dhsprogram.com/publications/publication-dhsq7-dhs-questionnaires-and-manuals.cfm
