# Supplementary figures and images for: Distance to available services for newborns at facilities in Malawi: A secondary analysis of survey and health facility data
Source: PLoS One. 2021 Jul 7;16(7):e0254083. doi: 10.1371/journal.pone.0254083 (PMC8263259; doi:10.1371/journal.pone.0254083)

S3 Table. Co-coverage of newborn care by place of birth


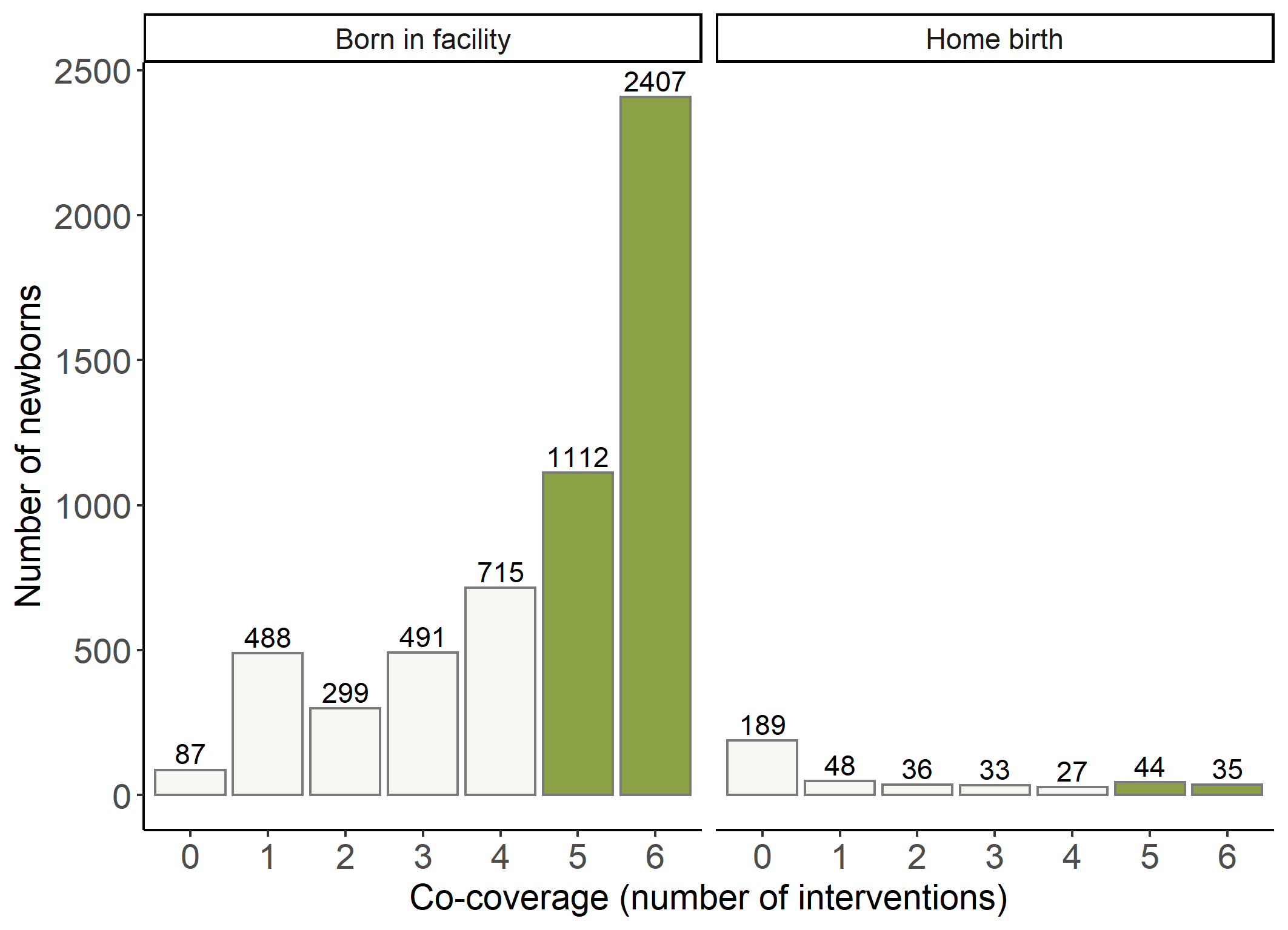

Supplement: S3 Table — (DOCX) [file pone.0254083.s003.docx]
