# Supplementary material for: Distance to available services for newborns at facilities in Malawi: A secondary analysis of survey and health facility data
Source: PLoS One. 2021 Jul 7;16(7):e0254083. doi: 10.1371/journal.pone.0254083 (PMC8263259; doi:10.1371/journal.pone.0254083)
Supplement: S4 Table — (DOCX) [file pone.0254083.s004.docx]

S4 Table. crude risk ratios

|  | Number of linked facilities | |
| --- | --- | --- |
|  | *RR* | *95%CI* |
| **5-10km Distance** |  |  |
| **Number of facilities (ref=none)** |  |  |
| One facility | 0.97 | 0.88,1.07 |
| Two or more facilities | 1.01 | 0.92,1.11 |
| **Service environment score (ref=lowest)** |  |  |
| No facility | 1.03 | 0.85,1.26 |
| Middle | **1.18** | **1.08,1.30** |
| Highest | **1.30** | **1.19,1.41** |
| **2-hour walk** |  |  |
| **Number of facilities (ref=none)** |  |  |
| One facility | 0.97 | 0.88,1.07 |
| Two or more facilities | 1.01 | 0.92,1.11 |
| **Service environment score (ref=lowest)** |  |  |
| No facility | **1.13** | **1.02,1.26** |
| Middle | **1.13** | **1.02,1.25** |
| Highest | **1.24** | **1.13,1.36** |
| **Home birth** | **0.31** | **0.24,0.41** |
| **Population density (ref=lowest density)** |  |  |
| Middle density | 1.03 | 0.95,1.13 |
| Most dense | **1.14** | **1.04,1.23** |
| **Wealth (ref=poorest)** |  |  |
| Poorer | 1.07 | 0.99,1.15 |
| Middle | **1.09** | **1.01,1.19** |
| Richer | **1.13** | **1.04,1.26** |
| Richest | **1.21** | **1.11,1.32** |
| **Maternal age at birth (ref=less than 20 years)** |  |  |
| 20-34 years | 0.99 | 0.93,1.06 |
| 35+ years | 1.00 | 0.91,1.09 |
| **Maternal education, secondary or higher** | **1.15** | **1.09,1.21** |
