# Supplementary material for: Distance to available services for newborns at facilities in Malawi: A secondary analysis of survey and health facility data
Source: PLoS One. 2021 Jul 7;16(7):e0254083. doi: 10.1371/journal.pone.0254083 (PMC8263259; doi:10.1371/journal.pone.0254083)
Supplement: S5 Table — (DOCX) [file pone.0254083.s005.docx]

S5 Table. Sensitivity analysis excluding home births

| **5-10km Distance** | | | | | |
| --- | --- | --- | --- | --- | --- |
| **Number of linked facilities** | | | **Service environment** | | |
|  | *ARR* | *95%CI* |  | *ARR* | *95%CI* |
| **Number of linked facilities (ref=none)** |  |  | **Service environment score (ref=lowest)** |  |  |
| One facility | 1.00 | 0.84,1.2 | No facility (within 5-10km) | 1.09 | 0.91,1.3 |
| Two or more facilities | 1.03 | 0.86,1.22 | Middle | **1.17** | **1.07,1.28** |
|  |  |  | Highest | **1.22** | **1.11,1.35** |
| **Population density (ref=lowest density)** |  |  | **Population density (ref=lowest density)** |  |  |
| Middle density | 1.02 | 0.94,1.11 | Middle density | 1.00 | 0.92,1.09 |
| Most dense | **1.08** | **1,1.17** | Most dense | 1.02 | 0.93,1.11 |
| **Wealth (ref=poorest)** |  |  | **Wealth (ref=poorest)** |  |  |
| Poorer | 1.06 | 0.98,1.14 | Poorer | 1.05 | 0.97,1.13 |
| Middle | 1.05 | 0.97,1.14 | Middle | 1.05 | 0.97,1.13 |
| Richer | 1.06 | 0.98,1.15 | Richer | 1.05 | 0.97,1.14 |
| Richest | 1.06 | 0.97,1.17 | Richest | 1.04 | 0.94,1.14 |
| **Maternal age at birth (ref=less than 20 years)** |  |  | **Maternal age at birth (ref=less than 20 years)** |  |  |
| 20-34 years | 1.00 | 0.93,1.06 | 20-34 years | 1.00 | 0.93,1.07 |
| 35+ years | 1.05 | 0.97,1.15 | 35+ years | 1.06 | 0.97,1.16 |
| **Maternal education, secondary or higher** | **1.09** | **1.03,1.16** | **Maternal education, secondary or higher** | **1.08** | **1.02,1.15** |
|  |  |  |  |  |  |
| Number of births | 5588 |  | Number of births | 5523 |  |
| Number of clusters | 837 |  | Number of clusters | 828 |  |
|  |  |  |  |  |  |
| **Travel time: 2-hour walk** | | | | | |
| **Number of linked facilities** | | | **Service environment** | | |
|  | *ARR* | *95%CI* |  | *ARR* | *95%CI* |
| **Number of linked facilities (ref=none)** |  |  | **Service environment score (ref=lowest)** |  |  |
| One facility | 0.96 | 0.87,1.05 | No facility (within 2hr walk) | **1.13** | **1.03,1.25** |
| Two or more facilities | 0.94 | 0.85,1.04 | Middle | **1.11** | **1.01,1.22** |
|  |  |  | Highest | **1.15** | **1.04,1.26** |
| **Population density (ref=lowest density)** |  |  | **Population density (ref=lowest density)** |  |  |
| Middle density | 1.04 | 0.95,1.13 | Middle density | 1.03 | 0.95,1.12 |
| Most dense | **1.11** | **1.02,1.22** | Most dense | 1.07 | 0.98,1.17 |
| **Wealth (ref=poorest)** |  |  | **Wealth (ref=poorest)** |  |  |
| Poorer | 1.05 | 0.98,1.13 | Poorer | 1.06 | 0.98,1.14 |
| Middle | 1.05 | 0.97,1.14 | Middle | 1.05 | 0.98,1.14 |
| Richer | 1.06 | 0.98,1.15 | Richer | 1.06 | 0.98,1.15 |
| Richest | 1.07 | 0.97,1.17 | Richest | 1.04 | 0.95,1.14 |
| **Maternal age at birth (ref=less than 20 years)** |  |  | **Maternal age at birth (ref=less than 20 years)** |  |  |
| 20-34 years | 0.99 | 0.93,1.06 | 20-34 years | 1.00 | 0.93,1.07 |
| 35+ years | 1.05 | 0.96,1.15 | 35+ years | 1.05 | 0.97,1.15 |
| **Maternal education, secondary or higher** | **1.09** | **1.03,1.16** | **Maternal education, secondary or higher** | **1.09** | **1.02,1.15** |
|  |  |  |  |  |  |
| Number of births | 5588 |  | Number of births | 5570 |  |
| Number of clusters | 837 |  | Number of clusters | 835 |  |
